# Bovine Mastitis Therapy at a Crossroads: Pharmacokinetic Barriers, Biofilms, Antimicrobial Resistance, and Emerging Solutions

**DOI:** 10.3390/ph19010175

**Published:** 2026-01-19

**Authors:** Alexandra Ban-Cucerzan, Adriana Morar, Emil Tîrziu, Iulia-Maria Bucur, Sebastian-Alexandru Popa, Kálmán Imre

**Affiliations:** 1 Faculty of Veterinary Medicine, University of Life Sciences “King Mihai I” from Timisoara, 300645 Timisoara, Romania; emiltarziu@usvt.ro (E.T.); iulia.bucur@usvt.ro (I.-M.B.); sebastian.popa@usvt.ro (S.-A.P.); kalmanimre@usvt.ro (K.I.); 2 Research Institute for Biosecurity and Bioengineering, University of Life Sciences “King Mihai I” from Timisoara, 300645 Timisoara, Romania

**Keywords:** bovine mastitis, pharmacokinetics/pharmacodynamics, intramammary drug delivery, biofilm-mediated tolerance, antimicrobial resistance, next-generation therapies

## Abstract

Bovine mastitis remains a major challenge in dairy production despite extensive antimicrobial use, with therapeutic failure increasingly attributed to factors beyond classical antimicrobial resistance (AMR). Growing evidence indicates that treatment inefficacy arises from the combined effects of pharmacokinetic/pharmacodynamic (PK/PD) constraints, biofilm-mediated tolerance, intracellular persistence, and the adaptive capacity of mastitis pathogens. Intramammary therapy is particularly limited by poor tissue penetration, episodic drug elimination via milk flow, and inactivation by milk components, frequently resulting in subtherapeutic exposure at the site of infection. These limitations are amplified in chronic and subclinical mastitis, where biofilms and intracellular reservoirs reduce antimicrobial susceptibility and promote relapse and resistance selection. This narrative review integrates current knowledge on pharmacokinetic and pharmacodynamic (PK/PD) barriers, microbial survival strategies, and antimicrobial resistance (AMR) mechanisms that underlie treatment failure in bovine mastitis. It critically evaluates conventional antimicrobial therapies alongside emerging approaches, including antimicrobial peptides, bacteriophages and endolysins, nanoparticle-based delivery systems, immunomodulators, CRISPR-guided antimicrobials, and drug repurposing strategies. Overall, available evidence highlights the potential of these approaches to enhance therapeutic durability, particularly in settings where biofilm formation, intracellular persistence, and resistance limit conventional treatment efficacy. By mapping research coverage across mastitis phenotypes and therapeutic outcomes, this review identifies key gaps in long-term efficacy and resistance mitigation and underscores the need for PK/PD-guided, biofilm-aware, and resistance-conscious strategies to support durable next-generation mastitis management.

## 1. Introduction

Bovine mastitis remains one of the most economically detrimental diseases in dairy production, leading to substantial losses through decreased milk yield, altered milk composition, increased culling rates, and elevated treatment and labor costs [1,2,3,4]. Beyond its direct impact on animal health and farm economics, mastitis also represents a One Health concern, as antimicrobial use in dairy cattle contributes to the emergence and dissemination of antimicrobial resistance (AMR) at the animal–food–human interface [5]. Over 140 microorganisms can cause mastitis; the disease’s multifactorial nature complicates both prevention and treatment. Despite the widespread use of intramammary antimicrobials over several decades, clinical and bacteriological cure rates remain inconsistent, particularly in infections caused by *Staphylococcus aureus*, *Escherichia coli*, and environmental streptococci [1,6,7,8,9,10]. Recurrence and chronicity continue to challenge practitioners, suggesting that current therapeutic strategies are insufficiently addressing the biological and pharmacological complexity of the disease. A growing body of evidence indicates that treatment failures are strongly linked to pharmacokinetic (PK) and pharmacodynamic (PD) constraints inherent to the mammary gland environment, including limited tissue penetration, rapid drug elimination through milk flow, and drug inactivation by milk components [6,11]. These PK/PD limitations frequently result in subtherapeutic drug exposure, contributing not only to poor cure rates but also to the emergence of AMR [12,13,14]. AMR in mastitis pathogens, including β-lactamase production, target modification, efflux pump activation, biofilm formation, and intracellular persistence, further reduce therapeutic effectiveness and complicate disease control [13,14,15,16].

In response to these limitations, considerable research has focused on the development of alternative therapeutic approaches. Emerging modalities such as antimicrobial peptides (AMPs), bacteriophages, endolysins, nanoparticle-based delivery systems, immunomodulatory agents, and CRISPR-based antimicrobials offer novel mechanisms of action that differ fundamentally from those of traditional antibiotics. Many of these approaches demonstrate superior tissue penetration, robust anti-biofilm activity, or reduced potential for selecting resistant bacterial populations.

However, the current scientific literature typically examines PK/PD limitations, AMR dynamics, and innovative therapies in isolation. As a result, the field lacks a mechanistically integrated perspective capable of guiding rational drug development. Therefore, the objective of this review is to synthesize these domains and provide a unified analysis of how emerging therapeutics can overcome the pharmacological and microbiological barriers that limit conventional intramammary antibiotics.

## 2. Microorganisms Associated with Bovine Mastitis

Bovine mastitis is predominantly an infectious inflammatory condition of the mammary gland caused by a diverse but relatively well-defined group of microorganisms, with bacteria accounting for the vast majority of clinical and subclinical intramammary infections [1,2]. From a practical and epidemiological standpoint, mastitis pathogens are commonly classified according to their Gram reaction and their mode of transmission as contagious or environmental, a framework that remains central to disease control and therapeutic decision-making. Gram-positive cocci constitute the dominant group worldwide, with *Staphylococcus aureus* representing a key pathogen in chronic and recurrent mastitis due to its ability to persist within mammary tissue and evade host defenses [17,18,19,20,21]. Coagulase-negative staphylococci, including *Staphylococcus chromogenes*, *S. epidermidis*, and *S. simulans*, are increasingly recognized as prevalent causes of subclinical mastitis, particularly in herds where traditional contagious mastitis has been effectively controlled. Streptococci are another major group, with *Streptococcus agalactiae* classically associated with contagious transmission during milking, whereas *Streptococcus uberis* and *Streptococcus dysgalactiae* are more commonly linked to environmental exposure [1,8,17,18,19,20,21]. Gram-negative bacteria play a particularly important role in acute and severe mastitis, with *Escherichia coli* being the most frequently isolated species and a leading cause of acute clinical cases, especially during early lactation. Other Gram-negative organisms, including *Klebsiella* spp., *Enterobacter* spp., *Citrobacter* spp., *Pseudomonas* spp., and *Proteus* spp., are typically associated with contaminated bedding, manure, and housing environments; although these infections may be transient, they are often accompanied by intense inflammatory responses driven by endotoxin release, resulting in severe local and systemic manifestations [1,8,17,18,19,20,21] Epidemiologically, contagious pathogens such as *Staphylococcus aureus*, *Streptococcus agalactiae*, and *Mycoplasma bovis* use the udder as their primary reservoir and spread mainly during milking, frequently leading to persistent intramammary infections and herd-level transmission, whereas environmental pathogens originate from the cow’s surroundings and cause opportunistic infections influenced by hygiene, housing conditions, and seasonal factors; in herds with effective control of contagious mastitis, environmental pathogens often predominate [1,18,22,23,24]. Although bacteria are the principal etiological agents, non-bacterial microorganisms also contribute to bovine mastitis, particularly in chronic or refractory cases. *Mycoplasma bovis* represents a major contagious pathogen in certain regions and is associated with poor therapeutic response and rapid dissemination within herds. Fungal mastitis, most commonly caused by *Candida* species, occurs sporadically and is frequently linked to prolonged or repeated antimicrobial therapy, while algal mastitis due to *Prototheca* species represents a particularly challenging condition characterized by chronic infection, lack of effective antimicrobial treatment, and frequent necessity for culling. Viral agents and chlamydia-like organisms have occasionally been associated with mastitis but are considered rare and of secondary importance [17,24,25,26,27].

Advances in diagnostic approaches may further support optimized mastitis therapy by enabling earlier detection and pathogen-specific intervention. Recent studies have demonstrated the utility of molecular assays, such as real-time PCR, for rapid identification of methicillin-resistant *Staphylococcus aureus*, as well as host-derived biomarkers, including interleukin-6, as potential indicators of *Mycoplasma bovis*-associated mastitis [28,29].

### Biological and Pathophysiological Characteristics of Mastitis Pathogens Relevant to Therapy

Beyond taxonomic identity and epidemiological classification, mastitis-associated pathogens share a set of biological and pathophysiological characteristics that consistently shape disease behavior and therapeutic response and are therefore used as a unifying framework throughout this review [1,6]. These characteristics include the intrinsic speed and pathological pattern of infection development, tissue and cell tropism, organizational strategies such as biofilm formation, the capacity for intracellular persistence, physicochemical properties of the bacterial cell envelope, the occurrence of mixed infections, and the production of microbial factors that interfere with antimicrobial activity [20,30,31]. Together, these traits determine how pathogens interact with the mammary gland environment, influence accessibility to antimicrobial agents, and promote persistence despite treatment [22,32]. Importantly, these characteristics do not act in isolation but combine to create heterogeneous infection states in which bacterial populations differ in localization, metabolic activity, and susceptibility [26,31]. As a result, therapeutic outcome is not dictated solely by pathogen identity or in vitro susceptibility but by the cumulative impact of these biological constraints on antimicrobial exposure and effectiveness [1,6].

## 3. Current Therapeutics and Their PK/PD Limitations

Intramammary antimicrobials constitute the primary therapeutic intervention for bovine mastitis [6]. Despite broad historical use and demonstrated in vitro activity, clinical outcomes remain variable and frequently suboptimal. Understanding the PK and PD barriers that limit drug performance is essential for contextualizing the need for innovative therapeutic strategies. Clinical implications of PK/PD failure include treatment failure, increased economic losses, and the need for dose optimization based on PK/PD modeling to improve cure rates and limit resistance [33,34,35,36,37].

### 3.1. Pharmacokinetic Challenges in Intramammary Therapy

Intramammary therapy is constrained by several PK challenges that arise from the unique anatomy and physiology of the bovine mammary gland. One of the most significant barriers is the blood–milk barrier, where tight junctions between mammary epithelial cells restrict passive drug movement and lead to compartmentalized distribution; consequently, drugs administered to one quarter exhibit minimal transfer to others, necessitating direct treatment of each affected gland [38]. Uniform drug distribution is further complicated by the gland’s alveolar and ductal structure, with retention influenced by factors such as milk yield, residual volume, and milking frequency, all of which contribute to variable persistence of intramammary drug residues and inconsistent therapeutic exposure [39,40]. Additionally, intramammary elimination occurs episodically during milking rather than continuously as in systemic circulation, rendering traditional PK models inadequate and driving the need for physiologically based approaches that account for this discontinuous clearance pattern [39,40]. Accurate pharmacokinetic assessment is further hindered by sampling limitations, as sampling intervals are tied to milking schedules, and whole-milk measurements do not necessarily represent drug concentrations at the true sites of infection [39,41]. Considerable inter-individual and inter-quarter variability, including differences in inflammation, milk production, and tissue permeability, compounds the difficulty of predicting drug behavior and optimizing dosing regimens [41,42]. These challenges underscore the importance of advanced PK/PD modeling and physiologically based simulations to improve dosage design, predict residue depletion, and ensure therapeutic success while maintaining food safety [39,40]. Formulation strategies such as optimized particle size and sustained-release systems offer additional opportunities to improve intramammary retention and enhance distribution within the gland [43].

### 3.2. Pharmacodynamic Limitations and Consequence Exposure Profiles

Bovine mastitis treatment faces significant PD challenges beyond PK, leading to frequent clinical failures. Key PD barriers include: insufficient drug exposure at infection sites, biofilm-mediated tolerance, intracellular persistence of pathogens, and the selection of resistant strains.

#### 3.2.1. Insufficient Exposure at the Site of Infection

Experimental PK/PD modeling studies illustrate the clinical relevance of insufficient drug exposure at the site of infection. PK/PD modeling shows that many intramammary antibiotic formulations fail to maintain drug concentrations above the minimum inhibitory concentration (MIC) for the required duration (T > MIC for beta-lactams, AUC/MIC for concentration-dependent drugs) [33,34,35,36,37]. This subtherapeutic exposure is a primary driver of treatment failure, as antibiotics may not reach effective levels within the mammary gland, especially in the presence of milk inactivation and rapid drug washout [1,6,44]. For commonly used intramammary antimicrobials such as rifaximin and cefquinome, therapeutic success depends on achieving adequate PK/PD targets. For example, rifaximin requires maintaining an AUC/MIC above 14,281.63 h for *Staphylococcus aureus* and 57.80 h for *Escherichia coli* to achieve high cure rates, with dosing intervals and concentrations significantly impacting outcomes [33,34]. Cefquinome’s efficacy depends on bacterial load and dosing, with PK/PD targets varying by pathogen and inoculum size; failure to meet these targets can reduce bactericidal effects and promote resistance [35,37].

#### 3.2.2. Biofilm-Mediated Tolerance

Biofilm formation is a central driver of treatment failure in bovine mastitis, enabling pathogens to persist despite appropriate antimicrobial therapy and contributing substantially to chronic and recurrent infections. Biofilms comprise structured bacterial communities embedded within an extracellular polymeric substance (EPS) matrix of polysaccharides, proteins, and extracellular DNA, which restricts antibiotic penetration and slows molecular diffusion, preventing bactericidal concentrations from reaching deeply embedded cells [15,45,46,47,48]. Within this matrix, gradients of oxygen and nutrients reduce bacterial metabolic activity, fostering dormant, highly tolerant subpopulations that exhibit drastically diminished susceptibility to antimicrobial agents [46,47,48,49,50]. As a result, biofilm-embedded cells can withstand antibiotic concentrations higher than those required to kill planktonic bacteria, levels that standard intramammary formulations cannot achieve, leading to persistent intramammary infections despite in vitro susceptibility [31,51,52]. Beyond limiting drug penetration, biofilms fundamentally distort PK/PD targets by increasing the effective antimicrobial exposure required for bacterial killing (e.g., T > MIC and AUC/MIC), as reduced metabolic activity, altered microenvironments, and persister cell formation shift these targets far beyond those achieved by standard intramammary dosing regimens [53,54]. The EPS matrix also impairs immune cell access and function, further protecting the bacterial community [15,46,47,48,55]. Biofilms additionally harbor persister cells, metabolically inactive variants capable of surviving antimicrobial exposure and reseeding infection after treatment ends, making them a major determinant of relapse and chronicity [46,48,50,51,52]. Clinically, biofilm-associated infections are strongly linked to therapeutic failure in mastitis caused by *Staphylococcus aureus* and coagulase-negative staphylococci, and they explain why some udder quarters fail to respond to antimicrobial therapy even when laboratory testing indicates susceptibility [15,50,51,52,56]. Given these multifaceted interactions between biofilm architecture, microbial physiology, and antimicrobial exposure, biofilms impose simultaneous pharmacokinetic and pharmacodynamic barriers that conventional intramammary therapies are poorly equipped to overcome. These barriers affect not only drug penetration and residence time within the mammary gland but also the effective antimicrobial activity at the cellular level, particularly against dormant or deeply embedded bacterial populations. To summarize these interrelated limitations, Table 1 provides an overview of the major PK and PD constraints imposed by biofilms and highlights their clinical implications for mastitis treatment.

#### 3.2.3. Intracellular Persistence

Intracellular persistence represents a critical survival strategy for mastitis pathogens, particularly *Staphylococcus aureus*, and is a major contributor to chronic and recurrent intramammary infections. Once internalized into mammary epithelial cells or neutrophils, *S. aureus* effectively evades both host immune defenses and antimicrobial exposure, creating a protected intracellular reservoir that can reseed infection even after clinically successful treatment. Many antibiotics used in mastitis therapy, including β-lactams and aminoglycosides, penetrate host cells poorly, resulting in subtherapeutic intracellular concentrations and limited bactericidal activity against internalized bacteria [57,58,59,60]. Within host cells, *S. aureus* can further enhance its survival by adopting a metabolically altered, non-replicating persister-like state that exhibits marked tolerance to antibiotics targeting active cellular processes [61,62,63]. This combination of reduced drug penetration, immune evasion, and metabolic dormancy explains why intracellular populations frequently survive therapy and subsequently trigger relapse, even when milk cultures appear temporarily negative [57,61]. Clinically, these intracellular reservoirs contribute to the low cure rates reported for *S. aureus* mastitis, often as low as 10–30% despite appropriate antimicrobial selection, and underscore the need for novel therapeutic approaches capable of targeting intracellular pathogens, including cell-penetrating antibiotics and agents that disrupt intracellular survival mechanisms [57,59,63,64,65].

#### 3.2.4. Selection of Resistant Strains

Exposure to sub-inhibitory antibiotic concentrations due to inadequate dosing, PK washout, or milk inactivation favors the selection of resistant subpopulations. This is exacerbated by incomplete treatment courses and the misinterpretation of transient clinical improvement as a cure, leading to multidrug-resistant (MDR) strains and persistent infections [14,16,44,66].

## 4. Antimicrobial Resistance in Mastitis Pathogens

AMR has become a central challenge in the management of bovine mastitis, undermining the effectiveness of commonly used intramammary antibiotics. Resistance patterns vary widely among pathogens, farms, and geographic regions, but several consistent trends have emerged. Understanding the molecular and ecological basis of resistance is essential for designing therapies capable of overcoming these mechanisms.

### 4.1. Molecular Mechanisms of Antimicrobial Resistance

Diverse and often redundant molecular mechanisms enable mastitis pathogens to withstand antimicrobial exposure during intramammary therapy. The most relevant mechanisms include enzymatic inactivation (e.g., β-lactamase production), target site modification, active drug efflux, and the dissemination of resistance determinants through genetic mobility and horizontal gene transfer.

#### 4.1.1. β-Lactamase Production

Mastitis pathogens employ a wide array of molecular mechanisms to survive antimicrobial exposure during intramammary therapy, with β-lactamase production representing one of the most widespread and clinically consequential forms of resistance. β-lactamases enzymatically hydrolyze the β-lactam ring of penicillins and cephalosporins, inactivating these drugs even when transient concentration spikes exceed the MIC, thereby undermining the efficacy of first-line intramammary treatments [67,68,69]. In staphylococci, the *blaZ* gene, present in multiple variants (A–D and the recently described F), encodes β-lactamase production, and its frequent localization on mobile genetic elements facilitates horizontal dissemination across species and within dairy herds [67,68,70]. Prevalence rates are strikingly high, with *blaZ* carriage reported in up to 91% of coagulase-negative staphylococci and similarly elevated detection in *Staphylococcus aureus* and Gram-negative mastitis pathogens [71,72,73,74,75]. In Gram-negative bacteria such as *Escherichia coli* and *Klebsiella* spp., extended-spectrum β-lactamases (ESBLs), including CTX-M, TEM, and SHV families, confer resistance to an even broader range of β-lactams, including third- and fourth-generation cephalosporins commonly used in veterinary medicine [71,72,73,74,75,76,77]. Clinically, β-lactamase production contributes to treatment failure, persistent intramammary infections, and the emergence of multidrug-resistant strains. Particularly concerning are advanced β-lactamase variants such as the newly reported *blaZ* variant F in *S. aureus*, which degrade a wider spectrum of β-lactams, including oxacillin, and often escape detection by routine phenotypic tests, further complicating therapeutic decision-making [67,68,70,74].

#### 4.1.2. Target Site Modification

Target site modification represents one of the most important molecular strategies by which mastitis-associated pathogens evade antimicrobial activity, enabling resistance across multiple drug classes. Alterations in penicillin-binding proteins (PBPs) are a major driver of β-lactam resistance, arising through point mutations, homologous recombination, or acquisition of alternative PBP genes such as *mecA*, which encodes the low-affinity PBP2a characteristic of methicillin-resistant *Staphylococcus aureus* [78,79,80,81,82,83]. Specific amino acid substitutions, including M341I in PBP2x or P409T in PBP1a, have been associated with high-level β-lactam resistance in pathogens such as *Streptococcus suis* and *Enterococcus faecium*, illustrating the functional impact of PBP remodeling on clinical outcomes [81,82,83,84]. Ribosomal target modification further contributes to antimicrobial resistance through methylation of 23S rRNA at A2058, catalyzed by Erm methyltransferases. This modification blocks the binding of macrolides, lincosamides, and streptogramin B (MLS) antibiotics, conferring high-level resistance; more than 50 erm variants have been identified, displaying either constitutive or inducible expression patterns [85,86,87,88,89]. Fluoroquinolone resistance, meanwhile, is largely driven by mutations in the quinolone resistance–determining regions of DNA gyrase (GyrA, GyrB) and topoisomerase IV (ParC, ParE), which reduce antibiotic affinity and impair drug–target interactions [90,91,92,93,94,95]. Accumulation of multiple mutations can result in high-level resistance and facilitate the rapid clonal expansion of resistant strains [90,93,94]. Collectively, these diverse forms of target site modification illustrate the adaptive versatility of mastitis pathogens and underscore the complexity of designing antimicrobial therapies capable of overcoming such entrenched resistance mechanisms.

#### 4.1.3. Efflux Pumps

Efflux pumps are major contributors to antimicrobial resistance in mastitis-associated bacteria, functioning as membrane protein complexes that actively expel antibiotics and toxic compounds to maintain intracellular drug concentrations below bactericidal levels. These systems play a central role in MDR across both Gram-negative and Gram-positive pathogens, particularly undermining the efficacy of macrolides, tetracyclines, fluoroquinolones, aminoglycosides, and several β-lactams. Among the major efflux pump families, the Resistance–Nodulation–Division superfamily is especially significant in Gram-negative organisms, with the AcrAB–TolC tripartite pump serving as a dominant determinant of intrinsic and acquired MDR in Enterobacteriaceae [96,97,98,99,100,101,102]. Additional efflux systems involved in mastitis pathogens include the Major Facilitator Superfamily (MFS), such as the NorA pump in staphylococci, as well as ATP-Binding Cassette (ABC), Small Multidrug Resistance (SMR), and MATE transporters [100,103]. These pumps exhibit either narrow or broad substrate specificity, frequently conferring resistance to multiple structurally unrelated antibiotic classes and thereby complicating therapeutic decision-making [96,99,100,104]. Overexpression driven by regulatory mutations or environmental stimuli can further elevate resistance levels, contributing to clinical treatment failure and facilitating the spread of MDR phenotypes [98,102,105]. Efflux activity often acts synergistically with other resistance mechanisms such as reduced membrane permeability or enzymatic drug inactivation resulting in cumulative and sometimes formidable resistance profiles [96,97,100]. Although no efflux pump inhibitors (EPIs) have yet reached clinical application, both natural compounds and synthetic molecules targeting pump components show promise in restoring antimicrobial susceptibility, and next-generation antibiotics such as omadacycline have been engineered specifically to evade efflux-mediated resistance [100,103,106,107,108,109].

#### 4.1.4. Genetic Mobility and Horizontal Gene Transfer

The spread of antimicrobial resistance in mastitis-associated bacteria is driven largely by genetic mobility and horizontal gene transfer (HGT), which enable resistance determinants to disseminate rapidly within and between bacterial species, particularly under selective pressure from prolonged or repeated antimicrobial use [110,111,112]. Mobile genetic elements (MGEs), including plasmids, transposons, and integrons, serve as the principal vehicles for this transfer, creating a dynamic genomic environment that accelerates the emergence of multidrug-resistant populations [110,113]. Plasmids, especially conjugative forms, are among the most potent vectors of resistance, carrying multiple antibiotic resistance genes (ARGs) and enabling their direct transmission across species and even genus boundaries [114,115,116,117,118,119]. These plasmids frequently act as hubs for ARG acquisition, incorporating additional resistance genes through insertion sequences and integron-mediated recruitment [120,121]. Transposons further enhance genetic mobility by moving resistance determinants between plasmids and chromosomes, facilitating recombination events that expand bacterial adaptability under antibiotic pressure [110,113,115,116,121,122,123,124]. Integrons, although not self-mobile, play a central role in capturing and expressing resistance gene cassettes; when embedded within plasmids or transposons, they contribute substantially to the dissemination of multidrug resistance, with class 1 integrons being particularly prominent in both clinical and agricultural settings [115,116,117,122,123]. Selective pressures including sustained antibiotic exposure, heavy metal co-contamination, and environmental stressors further accelerate the mobilization and retention of ARGs, promoting the persistence of resistant strains within herds and the surrounding environment [114,121,125,126,127]. The molecular and cellular mechanisms create a complex biological context that directly constrains the effectiveness of conventional mastitis therapies. Importantly, these mechanisms do not operate in isolation but interact with the pharmacological properties of available antimicrobials, influencing drug penetration, activity, and persistence within the mammary gland [30,31,128].

### 4.2. Antibiotics Currently Used in Mastitis Therapy

The primary antibiotic classes currently used for bovine mastitis are beta-lactams, aminoglycosides, tetracyclines, sulfonamides, lincosamides, and fluoroquinolones (Table 2). Selection depends on pathogen, resistance patterns, and local regulations. Rising resistance highlights the need for prudent, targeted use and ongoing development of alternative therapies.

#### 4.2.1. Beta-Lactams

Penicillins and cephalosporins remain the most widely used antibiotics for treating intramammary infections in dairy cattle. However, their clinical effectiveness is increasingly compromised by the widespread emergence of β-lactamase-mediated resistance among mastitis pathogens [131,132,133]. Ceftiofur (a third-generation cephalosporin) is particularly common in North America and Europe [6,16,129].

High rates of resistance are reported in key mastitis pathogens (Table 3). Resistance is often plasmid-mediated, facilitating rapid spread within and between herds [5,134]. At the same time, regional surveillance and field studies from Europe and South America, including Brazil and several European dairy production systems, have reported low or limited resistance to β-lactams in certain mastitis pathogens, particularly streptococci and subsets of *Staphylococcus aureus*, highlighting that resistance prevalence is highly context-dependent rather than uniform across dairy systems [135,136,137,138,139].

Clinical and epidemiological consequences of antimicrobial resistance in bovine mastitis are increasingly evident, with reduced cure rates and higher treatment failure, particularly affecting chronic or severe infections [140,141]. Beyond individual cow outcomes, AMR poses broader herd-level and public health risks, as resistance genes can disseminate horizontally to other bacterial populations, facilitating the emergence and persistence of multidrug-resistant strains within farm environments and potentially along the food chain [5,133,142]. Similar resistance patterns have also been reported in small ruminants, where recent data from sheep milk identified multidrug-resistant *Staphylococcus aureus*, *Streptococcus* spp., and *Enterococcus* spp. as dominant subclinical mastitis pathogens, further underscoring the broader epidemiological dimension of mastitis-associated AMR beyond bovine systems [143]. Of particular concern is the use of third-generation cephalosporins such as ceftiofur, which has been consistently associated with increased prevalence of extended-spectrum β-lactamase (ESBL)-producing bacteria in dairy herds, further compounding therapeutic challenges and elevating One Health risks [5,133,144]. Although combination therapy with β-lactamase inhibitors such as amoxicillin–clavulanate can partially restore antimicrobial efficacy, reports of resistance emerging even against inhibitor-based therapies underscore the accelerating erosion of available treatment options and the urgent need for novel interventions [76,131,145].

**Table 3:** β-lactam resistance rates and mechanisms in major bovine mastitis pathogens.

Pathogen	Resistance Mechanism	Resistance Rate (Penicillins/ Cephalosporins)	Key Genes	References
*Staphylococcus aureus*	β-lactamase (*blaZ*)	75–100%/50–57%	*blaZ*, *mecA*	[140,141,146,147,148]
*Escherichia coli*	ESBLs (*blaTEM*, *blaCTX-M*)	95–100%/70–97%	*blaTEM*, *blaCTX-M*	[5,76,134,142]
*Klebsiella* *pneumoniae*	ESBLs (*blaTEM*, *blaSHV*)	100%/62–75%	*blaTEM*, *blaSHV*	[5,76,134,142]

#### 4.2.2. Aminoglycosides

Aminoglycosides (gentamicin, amikacin, neomycin, streptomycin) and tetracyclines (such as oxytetracycline) remain commonly used in the treatment of bovine mastitis; however, rising resistance among major mastitis pathogens increasingly undermines their clinical value. *Staphylococcus aureus* exhibits substantial and geographically variable resistance, with gentamicin resistance ranging from 17% to 54% and tetracycline resistance from 28% to 66%, alongside high multidrug resistance levels exceeding 60% in several reports [149,150,151,152]. Among *Streptococcus* spp., resistance to tetracycline is notably high (59–86%), and neomycin resistance has reached 79% in some regions, whereas gentamicin resistance tends to be lower (2–16%) but is nonetheless present and increasing [153,154,155,156]. Although *Streptococcus agalactiae* remains highly susceptible to aminoglycosides in certain geographic areas, susceptibility patterns are inconsistent, and resistance is escalating globally [155,157]. Gram-negative pathogens such as *Escherichia coli* and *Klebsiella* spp. demonstrate particularly concerning profiles, with *E. coli* showing extremely high tetracycline resistance (89–100%) [76,158,159] and variable gentamicin resistance (7–47%) [160,161], while *Klebsiella* sp. isolates also display notable resistance to aminoglycosides (27%) and tetracyclines (36%) [76,160]. Consistent with these trends, multidrug resistance is widespread across mastitis isolates, ranging from 27% to 84% in published studies, frequently encompassing co-resistance to both aminoglycosides and tetracyclines [44,154,160].

#### 4.2.3. Lincosamides

Lincosamides such as pirlimycin and clindamycin play a targeted and increasingly important role in the management of bovine mastitis, particularly as multidrug-resistant pathogens become more prevalent in dairy herds. These agents are primarily effective against Gram-positive bacteria, including *Staphylococcus aureus* and *Streptococcus* spp., and are often selected for intramammary therapy when resistance to first-line antibiotics such as penicillins, cephalosporins, or tetracyclines is detected [16,130]. Their relatively narrow antibacterial spectrum allows for focused pathogen control with minimal disruption of beneficial udder microbiota, offering an advantage over broader-spectrum agents. Although resistance to lincosamides remains comparatively low, increased use has been associated with the emergence of resistant strains, underscoring the importance of antimicrobial stewardship [130]. Evidence indicates a positive association between patterns of pirlimycin use and increased resistance prevalence among mastitis-associated pathogens, reinforcing the need for judicious, evidence-based deployment of lincosamides within antimicrobial stewardship frameworks [130,162]. For this reason, lincosamides are typically reserved for cases in which first-line therapies have failed or where susceptibility testing confirms their necessity, thereby preserving their long-term clinical utility [16,130].

#### 4.2.4. Sulfonamides and Fluoroquinolones

Sulfonamides and fluoroquinolones occupy a selective but clinically relevant role in the treatment of bovine mastitis, particularly in cases where first-line therapies fail or when pathogens exhibit resistance to commonly used antibiotics. Sulfonamides, frequently administered in combination with trimethoprim, and fluoroquinolones such as enrofloxacin and marbofloxacin are generally reserved for infections involving Gram-negative organisms or for cases in which culture and susceptibility testing identify them as effective alternatives, in line with antimicrobial stewardship recommendations [7,162]. Sulfonamides combined with trimethoprim remain broadly effective against several mastitis-associated Gram-negative pathogens, with susceptibility often observed against opportunistic species such as *Proteus* and *Serratia* in some regional studies [163]. In clinical isolates of *Serratia marcescens* from bovine mastitis, approximately 72% of strains were susceptible to quinolones and sulfonamide–trimethoprim combinations, supporting their potential utility where first-line therapies fail [164]. Fluoroquinolones also demonstrate relatively strong activity in vitro, with norfloxacin showing approximately 66% sensitivity among mixed mastitis pathogens in field surveys, suggesting preserved effectiveness likely reflecting regulated or limited use in mastitis settings [163]. Despite these generally favorable profiles, both classes carry substantial risk for selection of resistance when overused, underscoring the need for targeted, evidence-based application and adherence to antimicrobial stewardship principles.

#### 4.2.5. Combination Therapies

Combination therapies such as β-lactams paired with aminoglycosides, β-lactamase inhibitors, or adjuncts like lactoferrin, herbal extracts, and nanoformulations are increasingly explored as strategies to counteract antimicrobial resistance and enhance treatment outcomes in bovine mastitis. Their effectiveness, however, is highly pathogen-dependent and varies considerably across drug pairings [6,7,165,166]. For instance, β-lactam–aminoglycoside combinations are widely used in empirical therapy and may offer synergistic activity against *Staphylococcus aureus*, although such benefits are not consistently observed across all pathogens or when β-lactams are combined with β-lactamase inhibitors [167,168]. Stronger synergy has been reported with immunomodulatory adjuncts: the addition of lactoferrin to penicillin G markedly increased cure rates in β-lactam-resistant *S. aureus* infections from 9.1% with penicillin alone to 45.5% with the combination, demonstrating the potential of biologically active adjuvants to restore antimicrobial efficacy [7,165,169]. In parallel, combinations involving antimicrobial peptides and non-antibiotic adjuvants have shown promising results, as exemplified by the synergistic activity of the peptide lynronne-1 with EDTA, which significantly reduced bacterial loads of diverse mastitis pathogens compared with monotherapy in experimental models [170]. Plant-based and nanoenhanced formulations contribute additional opportunities; herbal nanoemulsions such as *Achyrocline satureioides* exhibit synergy with aminoglycosides but not with β-lactams, highlighting selective enhancement of antimicrobial activity [167]. Similarly, Aloe vera–based formulations combined with low doses of cloxacillin or ceftiofur have shown promising pharmacokinetic profiles and potential therapeutic benefit, though further in vivo validation is required before clinical application [171]. Also, the rationale for combination therapies is supported by mechanistic evidence demonstrating that natural compounds, antimicrobial peptides, and other non-antibiotic agents can disrupt biofilm architecture, interfere with quorum sensing, and enhance antibiotic penetration, thereby restoring bacterial susceptibility to conventional antimicrobials [172]. Antibiotic-free combinations, such as bacteriocins used in conjunction with peptidoglycan hydrolases, further illustrate this principle and have demonstrated efficacy against both planktonic and biofilm-associated bacteria, including methicillin-resistant strains, although translational and regulatory challenges remain [173]. Clinical evidence further indicates that combination therapy does not universally result in improved outcomes. Randomized noninferiority trials have demonstrated that local intramammary penicillin treatment alone can be as effective as combined local and systemic therapy for mild to moderate mastitis caused by Gram-positive bacteria, suggesting that combination regimens may allow reduced systemic antibiotic exposure without compromising cure rates [174]. Beyond antimicrobial pairings, emerging combination strategies integrate nanoparticle-based drug delivery systems and immunotherapeutic or immunomodulatory interventions to address biofilm-associated tolerance and intracellular persistence [175,176]. Although these approaches are conceptually promising, most remain at an early experimental or clinical stage, with limited data on long-term efficacy, safety, residue profiles, and cost-effectiveness under field conditions [176,177].

## 5. Emerging and Experimental Therapeutic Options

The limitations of conventional therapies and the global pressure to reduce antibiotic use have stimulated the development of innovative therapeutic approaches for mastitis. These strategies aim either to improve the distribution and persistence of drugs in the mammary gland, to overcome bacterial resistance mechanisms, or to directly influence the biological processes involved in infection persistence, such as biofilm formation or immune dysfunction. This section summarizes the most relevant and promising emerging therapies, along with their potential for integration into next-generation treatments.

### 5.1. Antimicrobial Peptides (AMPs)

Among the most promising emerging alternatives to conventional antimicrobials are AMPs, naturally occurring molecules with broad-spectrum activity against key mastitis pathogens such as *Staphylococcus aureus* and *Escherichia coli*. AMPs exert rapid bactericidal effects primarily through membrane disruption, but they also interfere with biofilm development and can modulate host immune responses, offering a multifaceted therapeutic profile that addresses several limitations of traditional intramammary antibiotics [178,179]. In bovine intramammary in vivo contexts, endogenous antimicrobial peptides, particularly cathelicidins, have demonstrated significant antibacterial activity accompanied by attenuation of inflammatory responses, contributing to reduced bacterial burden and improved mammary gland homeostasis during mastitis [180,181]. AMPs provide several potential advantages, including activity against multidrug-resistant and biofilm-forming organisms, a relatively low propensity for resistance development, and additional immunomodulatory or wound-healing benefits [16,179]. However, their translation into clinical use remains limited by challenges such as high production costs, susceptibility to proteolytic degradation, potential cytotoxicity, and the scarcity of robust in vivo data in dairy cattle [179,180,182,183]. Although resistance to AMPs appears less common compared with conventional antibiotics, emerging reports highlight the need for continued monitoring and optimization of AMP-based therapies [179,180]. Together, these findings position AMPs as compelling candidates for next-generation mastitis therapeutics while underscoring the need for further refinement and clinical evaluation.

### 5.2. Bacteriophages and Endolysins

Bacteriophages and their lytic enzymes, endolysins, have emerged as highly specific and innovative alternatives to antibiotics for the treatment of bovine mastitis, particularly in the context of rising antimicrobial resistance. Phages provide precision targeting by infecting and lysing specific bacterial strains, including multidrug-resistant and biofilm-forming pathogens, while preserving beneficial microbiota [184,185,186,187,188]. In addition to direct bacteriolytic activity, bacteriophages have demonstrated strong antibiofilm effects against key mastitis-associated pathogens such as *Staphylococcus aureus* and *Escherichia coli*, mediated in part by phage-encoded depolymerases that disrupt the extracellular polymeric matrix; however, most evidence for these effects remains derived from in vitro or preclinical experimental models rather than controlled intramammary trials [189]. Endolysins, the cell wall–degrading enzymes encoded by bacteriophages, offer an even more stable and standardized approach, with strong activity against Gram-positive bacteria such as *Staphylococcus aureus* and enhanced ability to penetrate and disrupt biofilms, an essential advantage for managing chronic mastitis [186,188,190]. Recent advances in protein engineering have produced chimeric and modular endolysins with expanded host range and improved lytic activity [191,192,193], while encapsulation in nanoparticles or liposomes has shown promise in improving enzyme stability, delivery, and bioavailability within the milk-rich mammary environment [194,195]. Both phages and endolysins demonstrate a low propensity for resistance development due to their action on highly conserved cell wall structures, and several studies report synergistic effects when combined with antibiotics or other antimicrobials [184,185,195]. However, important limitations remain, including reduced stability in milk, inconsistent efficacy in vivo, limited clinical trial data in dairy cattle, and significant regulatory hurdles that currently restrict therapeutic adoption [193,196]. Nevertheless, ongoing innovations such as thermostable constructs, broad-spectrum engineered enzymes, and optimized delivery platforms underscore the considerable potential of phage-based and endolysin-based approaches as next-generation therapies for bovine mastitis [197,198].

### 5.3. Immunomodulatory Agents

Immunomodulatory interventions represent a complementary class of emerging therapies for bovine mastitis, targeting the host immune system rather than acting directly on pathogens. Since mastitis involves both bacterial infection and a dysregulated inflammatory response, these agents aim to optimize immune activation, improve bacterial clearance, and mitigate excessive inflammation, ultimately reducing reliance on traditional antibiotics. Several strategies are under investigation to modulate mammary immune function during intramammary infection.

#### 5.3.1. Toll-like Receptor (TLR) Agonists

Activation of TLR2 or TLR4 on mammary epithelial cells and phagocytes rapidly stimulates innate immune responses, driving the production of IL-1β, IL-6, TNF-α, and IL-8, and promoting early neutrophil recruitment. Preconditioning with ligands such as LPS or LTA has been shown to accelerate local immune activation and decrease bacterial burden in experimental mastitis [149,199,200,201,202].

#### 5.3.2. Cytokine Therapy (e.g., IL-8, GM-CSF, IFN-γ)

Exogenous cytokines enhance immune cell activation, trafficking, and microbial clearance. IFN-γ, in particular, reduces mastitis severity and shortens infection duration, though outcomes depend strongly on dosing regimen and timing [176].

#### 5.3.3. Anti-Inflammatory Biologics and Natural Immunomodulators

Compounds such as lactoferrin, gallic acid, and sodium alginate modulate inflammatory signaling and limit tissue damage without impairing bacterial killing [203,204,205]. Probiotics, including *Lactobacillus acidophilus* CRL2074, suppress TLR4-driven pro-inflammatory responses and help restore epithelial homeostasis [206,207].

The clinical application of immunomodulatory agents remains constrained by several important challenges, including variable efficacy depending on pathogen type, host immune status, and stage of disease, as well as the limited specificity of many currently available immunomodulatory interventions [30,176]. Host-related variability is exemplified by studies evaluating levamisole, which demonstrated reductions in somatic cell counts and macrophage numbers in subclinical mastitis but showed diminished efficacy in cows with high bovine leukemia virus proviral loads, underscoring the influence of underlying immune conditions on treatment outcome [208]. Additional limitations include the transient nature of immune activation induced by many immunomodulators, difficulties in achieving sustained or appropriately balanced immune responses, and the inherent complexity of the mammary gland immune environment, all of which can compromise consistent therapeutic benefit [30,209].

### 5.4. CRISPR-Guided Antimicrobials

CRISPR-guided antimicrobials represent a cutting-edge, precision-based approach, although still predominantly experimental, for targeting AMR bacteria in bovine mastitis, offering a fundamentally different strategy compared with traditional broad-spectrum antibiotics. These systems operate by directing the CRISPR–Cas complex toward specific resistance genes, either chromosomal determinants or AMR-carrying plasmids, leading to targeted DNA cleavage or functional inactivation of resistance traits, ultimately resulting in bacterial cell death or loss of resistance phenotypes [210,211,212,213,214,215,216,217]. This sequence-level precision enables the selective removal of resistant subpopulations with minimal disruption to the commensal microbiota and avoids the broad selective pressure imposed by conventional antimicrobials. CRISPR-guided systems have demonstrated the ability to suppress resistant bacterial populations and restore antibiotic susceptibility across various in vitro and in vivo experimental models [211,214,215,216,217]. Despite this promise, several major challenges hinder clinical translation, including the need to ensure CRISPR stability in the intramammary environment, optimize effective delivery vectors such as bacteriophages, plasmids, or nanoparticle carriers and address potential evolution of bacterial escape mechanisms [210,212,218,219]. Ethical and regulatory considerations remain significant barriers as well, particularly in food-producing animals where genome-targeting technologies must meet stringent safety, environmental, and consumer acceptance standards.

### 5.5. Drug Repurposing

The identification of new therapeutic applications for already approved or well-characterized pharmaceuticals has emerged as an efficient and cost-effective strategy for advancing innovative treatments for bovine mastitis, particularly in the context of rising antimicrobial resistance and increasing pressure to reduce antibiotic use. Because repurposed compounds possess established pharmacokinetic, pharmacodynamic, and toxicological profiles, they can progress more rapidly through preclinical evaluation and regulatory pathways, making them especially attractive for veterinary applications [183,195,204]. Several non-traditional agents have demonstrated promising anti-mastitis activity through mechanisms distinct from classical antibiotics. Disulfiram, originally developed for alcohol aversion therapy, exhibits potent activity against *Staphylococcus aureus*, including multidrug-resistant and biofilm-forming strains, by inducing oxidative stress, disrupting metal homeostasis, and impairing biofilm architecture; importantly, it has shown synergistic effects with conventional antibiotics in experimental models [220,221,222,223]. Gallium-based compounds act as iron mimetics that interfere with iron-dependent bacterial metabolism and inhibit biofilm formation, displaying broad-spectrum activity against both Gram-positive and Gram-negative pathogens, with evidence from in vitro studies, animal models, and early clinical investigations indicating slow resistance development [224,225,226,227,228]. Additional repurposing candidates include nonsteroidal anti-inflammatory drugs and ivermectin [229], which can disrupt bacterial membranes and modulate resistance mechanisms, as well as essential oils [230] that exert multi-target effects, including membrane destabilization and quorum-sensing inhibition; notably, these agents have demonstrated synergistic activity with antibiotics against biofilm-positive and resistant *S. aureus* strains. Collectively, repurposed agents offer several strategic advantages, including known safety profiles that reduce translational risk, the ability to enhance antibiotic efficacy and lower required doses when used as adjuvants, and novel mechanisms of action that target bacterial metabolism, virulence, and persistence rather than essential growth pathways, thereby potentially limiting resistance selection [231,232]. Nevertheless, important challenges remain, including the need for robust in vivo validation in dairy cattle, comprehensive milk-residue and food-safety assessments, and regulatory adaptation for veterinary use. Despite these hurdles, the mechanistic diversity and combinatorial potential of repurposed drugs underscore their strong promise as adjunctive or stand-alone components of next-generation mastitis treatment strategies [183,205]. Table 4 summarizes key non-antibiotic approaches, highlighting their mechanisms of action, principal advantages, current limitations, and level of supporting evidence.

## 6. Interpretation of Research Coverage Using a Literature Mapping Approach

**Figure 1:**
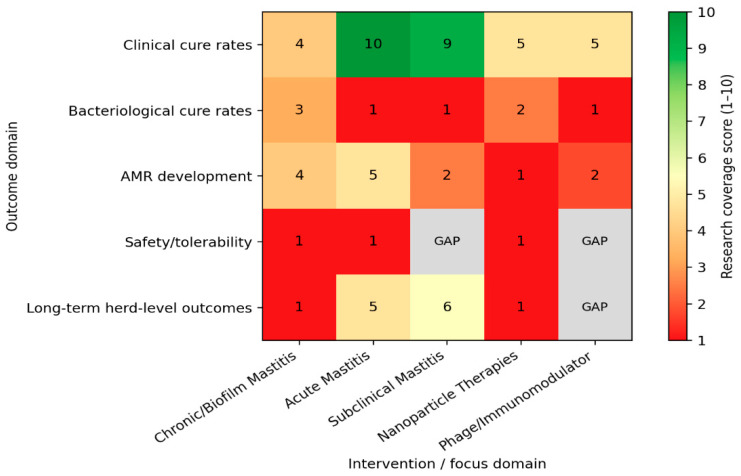
Heatmap illustrating the relative intensity of research coverage across mastitis types, therapeutic approaches, and key study outcomes. Higher scores indicate greater volume and maturity of published evidence, while lower scores highlight underexplored areas. “GAP” denotes the absence of meaningful long-term data [31,234,235,236,237,238,239,240,241,242,243,244,245,246,247,248,249].

An iterative narrative literature mapping approach was used to support the synthesis presented in this review and to construct the research coverage heatmap shown in Figure 1. An initial broad literature search was conducted using major scientific databases (Web of Science, Scopus, PubMed, and Google Scholar) to identify publications relevant to bovine mastitis therapy. As the conceptual structure of the review was developed, a representative core set of highly relevant publications, including review articles and original research, was selected based on thematic relevance rather than publication order. Following completion of the narrative synthesis, original experimental and clinical studies reporting primary data on mastitis therapy were extracted from this corpus and used for heatmap construction.

Studies included in the heatmap analysis were categorized according to predefined thematic dimensions, including mastitis type (acute, chronic, subclinical), therapeutic strategy (conventional antibiotics, combination therapies, antimicrobial peptides, bacteriophages or endolysins, nanoparticle-based approaches, immunomodulators, and other emerging interventions), and reported outcomes (clinical or bacteriological cure, biofilm-related endpoints, antimicrobial resistance indicators, and long-term or herd-level effects). Research coverage was estimated semi-quantitatively based on the relative frequency and depth of published evidence within each category, with higher coverage reflecting multiple or consistent independent investigations and lower coverage indicating sparse, preliminary, or context-specific data. Categories designated as “GAP” denote areas where meaningful or longitudinal evidence was insufficient to support robust conclusions. The research coverage heatmap was generated using IDE PyCharm Community Edition Python version 3.11.8. 

Within this analytical framework, the research imbalances highlighted in Figure 1 reflect key pharmacokinetic/pharmacodynamic (PK/PD) constraints and biological complexities that characterize bovine mastitis.

The predominance of studies focused on acute mastitis outcomes, particularly clinical and bacteriological cure rates, reflects therapeutic scenarios in which antibiotics are more likely to achieve sufficient exposure above the minimum inhibitory concentration (T > MIC) before extensive biofilm formation or intracellular persistence occurs. In contrast, the markedly lower research coverage observed for chronic, biofilm-associated, and subclinical mastitis corresponds to conditions where PK limitations such as poor tissue penetration, episodic drug elimination via milk flow, and inactivation by milk components severely restrict effective drug exposure at the site of infection. These constraints are further compounded by biofilm-mediated tolerance, which elevates the effective PK/PD targets far beyond those achieved by standard intramammary formulations.

The limited investigation of AMR outcomes and long-term herd-level effects, particularly for nanoparticle-based, phage, and immunomodulatory therapies, underscores a critical knowledge gap at the interface of PK/PD optimization and resistance suppression. Subtherapeutic exposure profiles, as suggested by the scarcity of AMR-focused studies, create ideal conditions for the selection and persistence of resistant subpopulations, especially within biofilm-protected niches. Notably, the near absence of long-term herd-level data for innovative therapies suggests that current research remains largely disconnected from One Health–relevant outcomes, such as resistance dissemination and microbiome perturbation.

Collectively, the heatmap emphasizes the need to reorient future mastitis research toward integrated PK/PD–biofilm–AMR frameworks, incorporating longitudinal designs and resistance-aware endpoints to ensure that emerging therapies translate into durable clinical and epidemiological benefits.

## 7. Concluding Remarks

Bovine mastitis therapy stands at a critical crossroads, where long-standing antimicrobial approaches increasingly fail to deliver durable clinical and bacteriological cures. As highlighted throughout this review, therapeutic inefficacy cannot be attributed solely to antimicrobial resistance but instead emerges from the convergence of multiple interdependent factors, including pathogen diversity, infection dynamics, pharmacokinetic and pharmacodynamic constraints, biofilm formation, intracellular persistence, and complex host–pathogen interactions within the mammary gland.

The marked heterogeneity of mastitis-associated microorganisms creates fundamentally different therapeutic scenarios that cannot be addressed by uniform treatment paradigms. These biological differences interact directly with the unique pharmacological environment of the mammary gland, where limited tissue penetration, episodic drug elimination via milk flow, and inactivation by milk components frequently result in subtherapeutic antimicrobial exposure at the site of infection. Such exposure profiles favor persistence, relapse, and the selection of resistant subpopulations, particularly in chronic and subclinical mastitis.

Biofilms and intracellular reservoirs further undermine classical PK/PD assumptions by elevating effective antimicrobial exposure thresholds beyond those achievable with standard intramammary formulations. In this context, in vitro susceptibility testing of planktonic bacteria provides an incomplete and often misleading predictor of clinical outcome. Integrating biofilm-aware and intracellular-targeted perspectives is therefore essential for accurately interpreting treatment failure and guiding the rational development of new therapeutic strategies.

Crucially, the translation of both conventional and next-generation mastitis therapies must occur within the non-negotiable constraints of food safety, regulatory approval, and One Health principles. Residue management, withdrawal periods, consumer acceptance, and environmental impact represent central determinants of whether a therapy can be responsibly deployed in food-producing animals. Approaches that reduce overall antimicrobial reliance, shorten treatment duration, or enhance targeted efficacy may offer regulatory and societal advantages, but only if supported by robust safety assessments, residue data, and field-relevant validation.

## Figures and Tables

**Table 1:** Biofilm-associated barriers and their impact on pharmacokinetics/pharmacodynamics, and clinical outcomes in bovine mastitis.

Biofilm Feature/Barrier	Impact on Pharmacokinetics (PK)	Impact on Pharmacodynamics (PD)	Clinical Consequences	References
EPS matrix (polysaccharides, proteins, eDNA)	Reduces drug penetration; slows diffusion into deeper microcolonies	Insufficient exposure prevents achieving effective T > MIC or AUC/MIC	Persistent infection despite susceptible MICs	[45,46,47]
Heterogeneous metabolic states	Not directly PK-related, but influences drug consumption and microenvironment	Dormant cells tolerate high antimicrobial concentrations; reduced killing	High rate of treatment failure and relapse	[47,48,50]
Altered pH and reduced oxygen tension	pH shifts can change drug ionization, affecting penetration.	Reduced drug activity for pH-sensitive antimicrobials.	Subtherapeutic effect even at adequate dosing	[15,46]
Reduced antibiotic diffusion gradients	Slower penetration increases required exposure time.	Biofilm cells survive concentrations up to 1000× MIC.	Recurrence after treatment cessation	[45,50]
Persister cell formation	Persisters minimally affected by PK exposure due to metabolic dormancy.	Survive therapy despite high drug concentrations.	Chronic, recurrent mastitis	[31,52]
Impaired immune access	Not a PK effect but limits host clearance.	Prevents synergistic immune–drug killing.	Failure of therapy even when drug reaches gland	[51,56]
Protection of intracellular reservoirs	Drugs fail to reach internalized bacteria.	PD failure due to insufficient intracellular activity.	Mixed acute–chronic infection profiles	[15,31]

Legend: PK, pharmacokinetics; PD, pharmacodynamics; EPS, extracellular polymeric substances; eDNA, extracellular DNA; MIC, minimum inhibitory concentration; T > MIC, time above the minimum inhibitory concentration; AUC/MIC, area under the concentration–time curve to MIC ratio. Clinical consequences refer to outcomes observed in biofilm-associated intramammary infections despite antimicrobial therapy.

**Table 2:** Main antibiotic classes and agents used in bovine mastitis.

Antibiotic Class	Common Agents Used	Typical Route	References
Beta-lactams	Penicillin G, ampicillin, amoxicillin, cloxacillin, cephapirin, ceftiofur, cephalexin, cefoperazone, ceftriaxone, cefotaxime, ceftazidime, cefquinome	Intramammary, systemic	[6,7,16,129,130]
Aminoglycosides	Gentamicin, amikacin, neomycin, streptomycin	Intramammary, systemic	[6,7,16,130]
Tetracyclines	Oxytetracycline, tetracycline	Intramammary, systemic	[6,7,16,130]
Sulfonamides	Trimethoprim-sulfamethoxazole, sulfonamides	Systemic	[6,7,130]
Lincosamides	Pirlimycin, clindamycin	Intramammary	[6,7,16]
Fluoroquinolones	Enrofloxacin, norfloxacin	Systemic	[6,130]

**Table 4:** Mechanisms, advantages, and limitations of emerging therapeutic strategies for bovine mastitis.

Therapeutic Strategy	Mechanism of Action	Key Advantages	Major Limitations	Evidence Status	References
Bacteriophages	Virus-mediated lysis of specific bacterial strains.	highly specific, minimal impact on microbiota; effective against MDR strains; potential biofilm activity.	stability reduced in milk; narrow host range; regulatory barriers; limited clinical trials.	In vitro, experimental animals; few bovine field studies	[184,185,187]
Endolysins	Enzymatic degradation of bacterial cell wall; strong activity against Gram-positive pathogens.	potent against *S. aureus;* penetrate biofilms; low resistance development; amenable to engineering (chimeras).	Stability challenges in milk; delivery limitations; few in vivo bovine studies.	Strong in vitro and rodent models; emerging bovine data.	[185,186,188]
Antimicrobial peptides	Membrane disruption; immune modulation; anti-biofilm activity.	active against MDR and biofilm-forming bacteria; reduced resistance development compared with conventional antibiotics; immunomodulatory benefits.	high production cost; proteolytic instability; potential cytotoxicity; limited bovine data.	Extensive in vitro studies; pilot in vivo models.	[16,179,180,233]
Nanoparticle delivery systems (e.g., liposomes, polymeric NPs, nanoemulsions)	Enhanced drug stability, targeted delivery, improved penetration. sustained release	Improves stability of enzymes/AMPs; enhances intramammary distribution; potentially lowers dose requirements;can disrupt biofilms	safety and milk residue concerns; production complexity; variable regulatory acceptance.	Rapidly expanding preclinical evidence; limited in vivo dairy studies.	[188,192,194]

Legend: MDR, multidrug-resistant; NPs, nanoparticles.

## Data Availability

No new data were created or analyzed in this study.

## Abbreviations

The following abbreviations are used in this manuscript:

AMR	Antimicrobial resistance
AMP/AMPs	Antimicrobial peptides
ARG/ARGs	Antibiotic resistance gene(s)
AUC	Area under the concentration
AUC/MIC	Area under the concentration to minimum inhibitory concentration ratio
ABC	ATP-binding cassette (efflux family)
CRISPR	Clustered regularly interspaced short palindromic repeats
Cas	CRISPR-associated protein(s)
EPS	Extracellular polymeric substances
DNAe	Extracellular DNA
ESBL	Extended-spectrum β-lactamase
CTX-M/TEM/SHV	ESBL enzyme families
EPI/EPIs	Efflux pump inhibitor(s)
HGT	Horizontal gene transfer
IFN-γ	Interferon gamma
IL	Interleukin
IL-1β/IL-6/IL-8	Interleukins 1β, 6, 8
LPS	Lipopolysaccharide
LTA	Lipoteichoic acid
MDR	Multidrug resistance
MFS	Major facilitator superfamily (efflux family)
MATE	Multidrug and toxic compound extrusion (efflux family)
MGE/MGEs	Mobile genetic element(s)
MIC	Minimum inhibitory concentration
MLS	Macrolide–lincosamide–streptogramin B
MRSA	Methicillin-resistant *Staphylococcus aureus*
CNS	Coagulase-negative staphylococci
NF-κB	Nuclear factor kappa B
NPs	Nanoparticles
PBPs	Penicillin-binding proteins
PBP2a	Low-affinity penicillin-binding protein (*mecA* product)
*mecA*	Methicillin resistance gene
*blaZ*	Staphylococcal β-lactamase gene
PD	Pharmacodynamics
PK	Pharmacokinetics
QRDR	Quinolone resistance–determining region
RND	Resistance–nodulation–division (efflux family)
AcrAB-TolC	RND tripartite efflux pump system
NorA	MFS efflux pump (staphylococci)
SMR	Small multidrug resistance (efflux family)
T > MIC	Time above the minimum inhibitory concentration
TLR	Toll-like receptor
TLR2/TLR4	Toll-like receptor 2/4
TNF-α	Tumor necrosis factor alpha
rRNA	Ribosomal ribonucleic acid
23S rRNA	23S ribosomal RNA
