# Correction: Ban-Cucerzan et al. Bovine Mastitis Therapy at a Crossroads: Pharmacokinetic Barriers, Biofilms, Antimicrobial Resistance, and Emerging Solutions. *Pharmaceuticals* 2026, *19*, 175

**DOI:** 10.3390/ph19030446

**Published:** 2026-03-10

**Authors:** Alexandra Ban-Cucerzan, Adriana Morar, Emil Tîrziu, Iulia-Maria Bucur, Sebastian-Alexandru Popa, Kálmán Imre

**Affiliations:** 1 Faculty of Veterinary Medicine, University of Life Sciences “King Mihai I” from Timisoara, 300645 Timisoara, Romania; emiltarziu@usvt.ro (E.T.); iulia.bucur@usvt.ro (I.-M.B.); sebastian.popa@usvt.ro (S.-A.P.); kalmanimre@usvt.ro (K.I.); 2 Research Institute for Biosecurity and Bioengineering, University of Life Sciences “King Mihai I” from Timisoara, 300645 Timisoara, Romania

In the original publication [1], the information on references [5,28,29,30,35,71,74,94,105,135,137,138,139,164,177,227,232] is not correct. The updated versions are as follows:

5. Velasco Garcia, W.J.; Araripe Dos Santos Neto, N.; Borba Rios, T.; Rocha Maximiano, M.; Souza, C.M.D.; Franco, O.L. Genetic basis of antibiotic resistance in bovine mastitis and its possible implications for human and ecological health. *Crit. Rev. Microbiol.* **2025**, *51*, 427–440. https://doi.org/10.1080/1040841x.2024.2369140.
28. Abd El-Razik, K.A.; Soror, A.H.; Sedky, D.; Fouad, E.A.; Arafa, A.A. Detection of methicillin-resistant *Staphylococcus aureus* (MRSA) from bovine subclinical mastitis in Egypt using real-time PCR. *Int. J. Vet. Sci.* **2024**, *14*, 188–195. https://doi.org/10.47278/journal.ijvs/2024.215.
29. Hayajneh, F.M.F.; Ahmed, Z.; Khatoon, A.; Saleemi, M.K.; Arshad, M.I.; Gul, S.T. Epidemiological investigations of *Mycoplasma bovis*—Associated mastitis in dairy animals along with analysis of interleukin-6 (IL-6) as a potential diagnostic marker. *Int. J. Vet. Sci.* **2023**, *13*, 120–126. https://doi.org/10.47278/journal.ijvs/2023.072.
30. Petzl, W.; Zerbe, H.; Günther, J.; Seyfert, H.-M.; Hussen, J.; Schuberth, H.-J. Pathogen-specific responses in the bovine udder. Models and immunoprophylactic concepts. *Res. Vet. Sci.* **2018**, *116*, 55–61. https://doi.org/10.1016/j.rvsc.2017.12.012.
35. Xiao, X.; Chen, X.; Yan, K.; Jiang, L.; Li, R.; Liu, Y.; Wang, M.; Wang, Z. PK/PD integration and pharmacodynamic cutoff of cefquinome against cow mastitis due to *Escherichia coli*. *J. Vet. Pharmacol. Ther.* **2022**, *45*, 83–91. https://doi.org/10.1111/jvp.13012.
71. Nery Garcia, B.L.; Dantas, S.T.A.; Da Silva Barbosa, K.; Mendes Mitsunaga, T.; Butters, A.; Camargo, C.H.; Nobrega, D.B. Extended-spectrum beta-lactamase-producing *Escherichia coli* and other antimicrobial-resistant Gram-negative pathogens isolated from bovine mastitis: A One Health Perspective. *Antibiotics* **2024**, *13*, 391. https://doi.org/10.3390/antibiotics13050391.
74. Campos, F.C.; Castilho, I.G.; Rossi, B.F.; Bonsaglia, É.C.R.; Dantas, S.T.A.; Dias, R.C.B.; Fernandes Júnior, A.; Hernandes, R.T.; Camargo, C.H.; Ribeiro, M.G.; et al. Genetic and antimicrobial resistance profiles of mammary pathogenic *E. coli* (MPEC) isolates from bovine clinical mastitis. *Pathogens* **2022**, *11*, 1435. https://doi.org/10.3390/pathogens11121435.
94. Arefin, M.S.; Mitu, M.J.; Mitu, S.Y.; Nurjahan, A.; Mobin, M.; Nahar, S.; Anjum, H.; Rahman, M.H. Mutational alterations in the QRDR regions associated with fluoroquinolone resistance in *Pseudomonas aeruginosa* of clinical origin from Savar, Dhaka. *PLoS ONE* **2025**, *20*, e0302352. https://doi.org/10.1371/journal.pone.0302352.
105. Lotfian, Z.; Nave, H.; Khalili, R.; Saffari, F. Contribution of efflux activity in resistance to antibiotics in *Escherichia coli* clinical isolates. *J. Infect. Chemother.* **2025**, *31*, 102725. https://doi.org/10.1016/j.jiac.2025.102725.
135. Thomas, V.; de Jong, A.; Moyaert, H.; Simjee, S.; El Garch, F.; Morrissey, I.; Marion, H.; Vallé, M. Antimicrobial susceptibility monitoring of mastitis pathogens isolated from acute cases of clinical mastitis in dairy cows across Europe: VetPath results. *Int. J. Antimicrob. Agents* **2015**, *46*, 13–20. https://doi.org/10.1016/j.ijantimicag.2015.03.013.
137. Monistero, V.; Barberio, A.; Cremonesi, P.; Castiglioni, B.; Morandi, S.; Lassen, D.C.K.; Astrup, L.B.; Locatelli, C.; Piccinini, R.; Addis, M.F.; et al. Genotyping and antimicrobial susceptibility profiling of *Streptococcus uberis* Isolated from a clinical bovine mastitis outbreak in a dairy farm. *Antibiotics* **2021**, *10*, 644. https://doi.org/10.3390/antibiotics10060644.
138. Dego, O.K.; Vidlund, J. Staphylococcal mastitis in dairy cows. *Front. Vet. Sci.* **2024**, *11*, 1356259. https://doi.org/10.3389/fvets.2024.1356259.
139. Majumder, S.; Sackey, T.; Viau, C.; Park, S.; Xia, J.; Ronholm, J.; George, S. Genomic and phenotypic profiling of *Staphylococcus aureus* isolates from bovine mastitis for antibiotic resistance and intestinal infectivity. *BMC Microbiol.* **2023**, *23*, 43. https://doi.org/10.1186/s12866-023-02785-1.
164. Moreira, G.; Pinho, L.; Mesquita, J.R.; Silva, E. *Serratia marcescens* isolates from bovine mastitic milk: antimicrobial resistance and virulence features. *Antibiotics* **2025**, *14*, 892. https://doi.org/10.3390/antibiotics14090892.
177. Debruyn, E.; Ghumman, N.; Peng, J.; Tiwari, H.; Gogoi-Tiwari, J. Alternative approaches for bovine mastitis treatment: A critical review of emerging strategies, their effectiveness and limitations. *Res. Vet. Sci.* **2025**, *185*, 105557. https://doi.org/10.1016/j.rvsc.2025.105557.
227. Cordeiro Gomes, F.D.; Ferreira Alves, M.C.; Alves Júnior, S.; Medina, S.H. Bactericidal metal–organic gallium frameworks-synthesis to application. *Mol. Pharm.* **2025**, *22*, 638–646. https://doi.org/10.1021/acs.molpharmaceut.4c01253.
232. Wang, Z.; Wang, J.; Shen, H. Evaluation of the therapeutic effect of a fly maggot antimicrobial peptide in a *Staphylococcus aureus*-induced mouse mastitis model. *Open Vet. J.* **2025**, *15*, 2540–2550. https://doi.org/10.5455/ovj.2025.v15.i6.26.

The authors state that the scientific conclusions are unaffected. This correction was approved by the Academic Editor. The original publication has also been updated.
